# Microbial and genetic-based framework identifies drug targets in inflammatory bowel disease

**DOI:** 10.7150/thno.59196

**Published:** 2021-06-01

**Authors:** Zhihan Wang, Kai Guo, Pan Gao, Qinqin Pu, Ping Lin, Shugang Qin, Na Xie, Junguk Hur, Changlong Li, Canhua Huang, Min Wu

**Affiliations:** 1 West China School of Basic Medical Sciences & Forensic Medicine, Sichuan University, Chengdu, Sichuan 610041, China.; 2 Department of Biomedical Sciences, School of Medicine and Health Sciences, University of North Dakota, Grand Forks, ND 58202, USA.; 3 Department of Neurology, University of Michigan, Ann Arbor, MI 48109, USA.; 4 Medical Research Institute, Wuhan University, Wuhan 430071, China.; 5 State Key Laboratory of Trauma, Burns and Combined Injury, Institute of Surgery Research, Daping Hospital, Army Medical University, Chongqing 400038, China.; 6 State Key Laboratory of Biotherapy and Cancer Center, West China Hospital, Sichuan University, and Collaborative Innovation Center for Biotherapy, Chengdu, Sichuan 610041, China.

**Keywords:** Inflammatory bowel disease, cyclic GMP-AMP synthase (cGAS), host transcriptome-microbiome interaction, drug repurposing, brefeldin-a

## Abstract

**Rationale:** With increasing incidence and prevalence of inflammatory bowel disease (IBD), it has become one of the major public health threats, and there is an urgent need to develop new therapeutic agents. Although the pathogenesis of IBD is still unclear, previous research has provided evidence for complex interplays between genetic, immune, microbial, and environmental factors. Here, we constructed a gene-microbiota interaction-based framework to discover IBD biomarkers and therapeutics.

**Methods:** We identified candidate biomarkers for IBD by analyzing the publicly available transcriptomic and microbiome data from IBD cohorts. Animal models of IBD and diarrhea were established. The inflammation-correlated microbial and genetic variants in gene knockout mice were identified by 16S rRNA sequences and PCR array. We performed bioinformatic analysis of microbiome functional prediction and drug repurposing. Our validation experiments with cells and animals confirmed anti-inflammatory properties of a drug candidate.

**Results:** We identified the DNA-sensing enzyme cyclic GMP-AMP synthase (cGAS) as a potential biomarker for IBD in both patients and murine models. cGAS knockout mice were less susceptible to DSS-induced colitis. cGAS-associated gut microbiota and host genetic factors relating to IBD pathogenesis were also identified. Using a computational drug repurposing approach, we predicted 43 candidate drugs with high potency to reverse colitis-associated gene expression and validated that brefeldin-a mitigates inflammatory response in colitis mouse model and colon cancer cell lines.

**Conclusions:** By integrating computational screening, microbiota interference, gene knockout techniques, and *in vitro* and *in vivo* validation, we built a framework for predicting biomarkers and host-microbe interaction targets and identifying repurposing drugs for IBD, which may be tested further for clinical application. This approach may also be a tool for repurposing drugs for treating other diseases.

## Introduction

Inflammatory bowel disease (IBD), including ulcerative colitis (UC) and Crohn's disease (CD), is a complex chronic inflammatory disease, typically manifested with diarrhea and weight loss. Mucosal inflammation in CD can affect any portion of the gastrointestinal tract, while UC is restricted to the colon. IBD has become an urgent public health threat, affecting 1.6 million Americans with 70,000 new cases each year and more than 3.5 million people worldwide 1. To date, the etiology of IBD remains incompletely understood though it is linked to genetic susceptibility, immunoregulatory factors, microbiome imbalance, and environmental factors 2.

Transcriptome analysis and microbiota profiling have emerged as promising approaches to assess host gene expression and survey microbiota composition quantitatively 3. Understanding of host-microbiome interactions in IBD pathogenesis is a critical avenue for therapeutic development. Genome-wide association studies (GWAS) have identified approximately 240 driver genes of IBD through analyzing a large number of patients 4. Many of these genes are associated with key immunological pathways, such as host-microbial interactions, innate immunity, adaptive immunity and autophagy 5. For example, NOD2-associated dysregulation of microbial sensing contributes to microflora dysbiosis 6 and CARD9 impacts colitis by altering gut microbiota metabolism 7. ATG16L1 affects autophagy in Paneth cells and goblet cells and regulates intestinal inflammation in an IL-22-dependent manner 8. Moreover, NOD2 and ATG16L1 are required for human commensal *Bacteroides fragilis*-mediated protection from colitis 9. Intestinal inflammation in *Card9^-/-^* mice is attenuated after inoculation of *Lactobacillus* strains 7. As part of the Integrative Human Microbiome Project (iHMP), the IBD Multi'omics database (IBDMDB) is the first integrated study supporting a systems-level understanding of gut microbiome in IBD dynamics 10. Altogether, the interactions between host and enteric microbiota may guide rapid development of treatments to improve intestinal health.

Innate immunity plays a vital role in the homeostasis of the intestine during the pathogenesis of IBD. Cyclic GMP-AMP synthase (cGAS) is a double-stranded DNA sensor and is essential for eliciting immune responses 11. cGAS recognizes the DNA from various invading microbes to activate the adaptor, stimulator of the interferon genes (STING), and induce type I interferon (IFN) secretion following phosphorylation of interferon regulatory transcription factor 3 (IRF3) by TANK binding kinase 1 (TBK1), and other pro-inflammatory cytokine production through NF-κB nuclear translocation 11. Intense research in recent years has implicated that cGAS signaling plays a role in the initiation and pathogenesis of autoimmune diseases, cancers, Aicardi-Gourtières syndrome (AGS), skin cancer, acute pancreatitis, and sepsis 12. Although the role of STING in regulating intestinal inflammation 13 and tumorigenesiss 14 in mice has been reported, the effect of cGAS on commensal microbiota in intestinal homeostasis remains to be determined.

Application of current IBD drugs is severely limited by low efficacy, side effects or intolerance, and particularly a drastic loss of response after long-term use 15. Since the discovery and development of new medications is time-consuming and labor-intensive, drug repurposing or drug repositioning becomes an attractive strategy for identifying new uses of 'old' drugs to potentially treat many debilitating diseases 16. For instance, denosumab is an antibody to TNFSF11 and has been used in osteoporosis of postmenopausal women 16. Since the TNFSF11 genetic variant was found to be associated with CD by GWAS, denosumab has been repurposed for treating CD and completed phase 1/2 trials in 2019 17. A large-scale computational method 18 demonstrates the potency to reverse disease signatures via drug discovery based on the Library of Integrated Network-Based Cellular Signatures (LINCS), a drug perturbation database maintained by the Broad Institute 19. The Connectivity Map Linked User Environment (CLUE) platform of LINCS is used to indicate treatment possibility of IBD by analyzing relationships between drugs and differentially expressed genes (DEGs) 20.

Here, we established a novel microbiota and genetic-based framework using a system bioinformatics approach based on host-microbiome interactions and laboratory studies. We identified cGAS as a potential biomarker in IBD, and knockout of cGAS in mice attenuated the inflammatory response. Next, we predicted IBD candidate therapeutics and validated the efficacy of the top candidate, brefeldin-a (BFA, a membrane protein transporter inhibitor), using colon cancer cell lines and mouse models. Our strategy may be applied to potentially reposition candidate drug targets and drugs, promoting drug discovery and medical improvement for other diseases.

## Methods

### Integrated Microbiome and Host Transcriptome Analysis

Publicly available RNA-seq and microbiome data on human IBD were collected from IBDMDB (http://ibdmdb.org) 10 (Table S1) and imported into R software version 4.0.2 21. The RNA-seq data were then normalized using a regularized log (rlog) transformation implemented in the DESeq2 package 22. Principal component analysis (PCA) of the host RNA-seq profile based on variance stabilizing transformation data was used to examine the overall similarity among all groups.

Only data from patients diagnosed as UC and non-IBD were used for integrated analyses, and a total of 4,811 DEGs were identified from the RNA-seq data with the adjusted *p-value* < 0.01 cutoff. Relative abundances of bacteria were calculated for microbiome data. Bacteria were filtered out with a relative abundance value < 0 in only three samples or less, and 169 bacteria were found for the following analysis.

We used two datasets from 43 common patients for the integrative analysis. Two-way orthogonal partial least squares (O2PLS), a method for pair-wise integration of multi-omics data, was used to identify novel key pathways and biomarkers that strongly correlated with the disease progression among different data matrix projects 23. The loading values for the joint covariance part from the O2PLS analysis results were extracted to find highly inter-associated genes and bacteria. Reactome 24 functional enrichment analysis for the top 200 genes (around 5% of the total DEGs) with the highest loading values was performed with the richR (https://github.com/hurlab/richR) package.

### Mice

Wild-type (WT) C57BL/6NHsd mice were obtained from ENVIGO (Indianapolis, IN). *Cgas^tm1a(EUCOMM)Hmgu^* mice (*Cgas*^-/-^ or cGAS KO mice) were kindly provided by Dr. Charlie Rice (Rockefeller University, NY) 25. All mice were housed in the animal facility at the University of North Dakota (UND) and treated according to guidelines approved by the Institutional Animal Care and Use Committee (IACUC) of UND. The age- and gender-matched mice (8-12 weeks old) were randomly allocated to different experimental groups based on their genotypes.

### Mice Treatments

The experimental model of acute colitis induced by dextran sulfate sodium (DSS, 3% wt/vol, 36,000-50.000 kD; MP Biomedicals, LLC, Aurora, OH) was administered in drinking water *ad libitum* for seven days, following four days of normal distilled water. Control mice were fed with distilled water for 11 days. Daily monitoring of mouse body weight, survival rate, and disease activity index (DAI), with a score of 1-4, including three parameters: weight loss, fecal blood, and stool consistency (0: no weight loss, no blood, normal stool; 1: 1-5% weight loss; 2: 5-10% weight loss, visible blood in the stool, loose stool; 3: 10-20% weight loss; 4: >20% weight loss, gross bleeding, diarrhea). Mice were sacrificed to collect intestine tissue and feces for further analysis at day 9. Colon lengths were also measured at autopsy. Fecal samples were collected for microbiome analysis.

For the diarrheal model induced by antibiotic treatment, a broad-spectrum antibiotic cocktail or individual antibiotic (5 mg/mL streptomycin, 0.5 mg/mL vancomycin, 1 mg/mlL colistin, and 1 mg/mL Ampicillin) (Sigma-Aldrich, St. Louis, MO) was added to drinking water for five days until analysis, as described previously 26. Streptomycin (STR) is a broad-spectrum antibiotic inhibiting both gram-positive and gram-negative bacteria, vancomycin (VAN) targets gram-positive bacteria, colistin (COL) targets *Enterobacteriaceae*, and ampicillin (AAM) targets gram-positive and some gram-negative bacteria 27.

### Myeloperoxidase (MPO) Assay

Colonic MPO levels were measured to determine the degree of neutrophil infiltration. The colon was weighed and homogenized in 50 mM hexadecyl trimethyl ammonium bromide (HTAB) - 50 mM potassium phosphate (pH 6.0) buffer. The homogenate was centrifuged at 13,400 × g for 6 min at 4 °C. The supernatant was collected or restored at -80 °C until use. 7 μL of tissue homogenate was mixed with 200 μL of reaction buffer (16.7 mg/mL o-dianisidine dihydrochloride, 50 mM potassium phosphate buffer [pH 6.0], 0.0006% H_2_O_2_) and was read at 450 nm using a spectrophotometer at 30 s intervals as previously performed 28.

### Enzyme-linked Immunosorbent Assay (ELISA)

Colonic intestinal tissue lysed with radiation immunoprecipitation (RIPA) buffer (Thermo Scientific, Waltham, MA) for cytokine analysis. The lysate was centrifuged at 15000 × g for 10 min, and then the supernatant was collected. Paired (capture and detection) antibodies and standard recombinant mouse IL-1β (eBioscience, San Diego, CA), TNF-α (eBioscience), and IFN-β (Invitrogen, Waltham, MA) were used to determine cytokine concentrations following the manufacturer's instructions 29.

### Immunoblotting

Mouse colon biopsies were freeze-thawed in RIPA buffer with protease and phosphatase inhibitor cocktail (ThermoFisher Scientific). The supernatant's protein concentration was quantified using the Pierce BCA protein assay kit (ThermoFisher Scientific). We resuspended the samples in 5X loading buffer (ThermoFisher Scientific) and heated to 100 ℃ for 10 min. An equal amount of samples was resolved by SDS-PAGE gel electrophoresis and transferred to the nitrocellulose membranes (Cytiva Amersham, LLC, Marlborough, MA). After blocked in TBS-T buffer supplemented with 5% blotting-grade non-fat milk at room temperature for 1.5 h, the membrane was subsequently incubated with antibodies against cGAS (D3O8O, Cell Signaling Technology [CST], Danvers, MA), STING (D2P2F, CST), p-TBK1 (D52C2, CST), TBK1 (D1B4, CST), TNF-α (D2D4, CST), NF-κB (D14E12, CST), Casp-1 (pro, sc-56036, Santa Cruz Biotechnology, Dallas, TE; cleaved, D57A2, CST), IL-1β (pro, D3H1Z, CST; cleaved, 52718S, CST), and β-actin (sc-517582, Santa Cruz), followed with the appropriate HRP-conjugated secondary antibody (Invitrogen) incubation, and detected by enhanced chemiluminescence (Cytiva Amersham) 30.

### Quantitative Polymerase Chain Reaction (qPCR)

Total RNA was extracted from mouse colon tissue homogenates with TRIzol reagent (Invitrogen), reverse transcribed into cDNAs by the Superscript III kit (11752, Invitrogen), and real-time PCR was performed using SYBR Green Master Mix (A25742, Applied Biosystems, LLC, Marlborough, MA) on the CFX Connect real-time PCR detection system (Bio-Rad, Hercules, California) as previously described 31. The primer sequences are as follows: *Cgas* F: 5'- CCACTGAGCTCACCAAAGAT-3'; *Cgas* R: 5'- CAGGCGTTCCACAACTTTATTC-3'; *Sting* F: 5'- GGTCACCGCTCCAAATATGTAG-3';* Sting* R: 5'- CAGTAGTCCAAGTTCGTGCGA-3';* Ifn-β* F: 5'- TCCGAGCAGAGATCTTCAGGAA-3';* Ifn-β* R: 5'- TGCAACCACCACTCATTCTGAG-3';* Ifit-1* F: 5'- CGTAGCCTATCGCCAAGATTTA-3';* Ifit-1* R: 5'- AGCTTTAGGGCAAGGAGAAC-3'; *Gapdh* F: 5'- TTGCTGTTGAAGTCGCAGGAG-3';* Gapdh* R: 5'- TGTGTCCGTCGTGGATCTGA -3'. These genes were normalized to the housekeeping gene *Gapdh* using 2^- ΔΔCT^ method.

### Histology and Immunofluorescence

Colon samples were extracted and washed with cold PBS, then immediately fixed with 10% formalin for 24 h and embedded in paraffin. 5-μm histological sections were deparaffinized and stained with hematoxylin and eosin (H&E) or Periodic acid-Schiff (PAS) and analyzed by light microscopy (Nikon Eclipse 80i). Histological assessment of colitis was performed with blinded genotypes as previously described 32, 33. The score criteria (0-3) included monocyte infiltration, epithelial injury, polymorphonuclear cell filtration, and crypt hyperplasia.

For immunofluorescence analysis, the mouse colon was immersed in the optimal cutting temperature (OCT) medium mold, snap-frozen in liquid nitrogen, and stored at -80 °C. 4 μm frozen sections were fixed in pre-chilled acetone for 5 min, stained with primary antibody MUC5AC (sc-21701, Santa Cruz), and then incubated with fluorescent-labeled secondary antibody (R&D Systems, Minneapolis, MN). DNA was stained with 20 µg/mL 4',6'-diamidino-2-phenylindole (DAPI, Invitrogen) 34. Zeiss LSM-510 Meta Confocal Microscope was used to obtain fluorescent images.

### Fecal DNA Extraction and 16S rRNA Library Preparation

According to the manufacturer, bacterial genomic DNA was extracted from approximately 0.2 g of frozen fecal samples using the QIAamp Fast DNA Stool Mini Kit (51604, Qiagen, Hilden, Germany). As previously described 35, the V3 and V4 hypervariable regions of the 16S rRNA gene were amplified using the following primers: F 5'-TCGTCGGCAGCGTCAGATGTGTATAAGAGACAGCCTACGGGNGGCWGCAG-3', R 5'-GTCTCGTGGGCTCGGAGATGTGTATAAGAGACAGGACTACHVGGGTATCTAATCC-3', and incorporated to Illumina (San Diego, CA) adapters and indexing barcodes. The amplified, barcoded products were purified using AMPure XP beads (Beckman Coulter, Indianapolis, IN) and pooled and sequenced on the Illumina MiSeq platform.

### 16S rRNA Sequencing Analysis

The raw pair-end sequencing data in FASTQ files were first assessed for quality by FastQC (version 0.11.3) 36 and filtered with Trimmomatic (version 0.36) 37 to remove low-quality reads (threshold quality < 30). Trimmed reads were assessed again for quality by FastQC. Taxonomy classification for trimmed reads was performed by Kraken 2 (version 2.0.8-beta) 38 based on the MiniKraken2_v1 database (version 201904), and sample report files were generated. Alpha diversity metrics based on bacterial counts at the genus level were performed using the estimate richness function within the phyloseq package (version 1.32.0) 39 and Shannon and Simpson indexes to measure the richness of the communities within samples. Principal Coordinates Analysis (PCoA) plot using Bray-Curtis dissimilarity was implemented based on bacterial counts at the genus level to reveal the differences between various groups. The 'Adonis' feature from vegan (version 2.5-6) was used to assess whether sample grouping by metadata factor accounted for inter-sample differences (https://CRAN.R-project.org/package=vegan). A significance *P*-value was generated by comparing that obtained by R^2^ to that obtained from 1000 random data permutations. Treemaps were generated from the genus percentage in report files by the treemapify package (version 2.5.3) (https://CRAN.R-project.org/package=treemapify) to show the relative abundance between different groups. Differential abundance analysis was performed using the DESeq2 package (version 1.28.1) with *p* < 0.05 and |*log2Fold Change*| > 1 based on bacterial counts at the genus level, and adjusted *p* < 0.05 at the species level 22. The taxon sets for TSEA were downloaded from MicrobiomeAnalyst, designed to predict biologically or ecologically meaningful patterns from public datasets and known microbial signatures 40. The TSEA of biologically meaningful taxon sets were performed with the richR package for predictive potential microbial targets to develop effective therapies.

### Relative Gene Expression Analysis by PCR Arrays

The real-time PCR primer sets of 85 genes (Table S7) were coated on 96-well plates (Eurofins Genomics) following the manufacturer's instructions, optimized according to the mouse RT^2^ Profiler PCR Array (PAMM-169Z, Qiagen) and relevant literature 41. After normalizing the three housekeeping genes (*Eef2*, *Gapdh,* and *Tbp*), the qPCR results were expressed as relative fold change over the WT control. The detection limit was 40 Ct. All experiments were performed in three biological replicates. The mouse transcriptomes were clustered according to genotype. Volcano plots and the heatmap were generated in the R package ggplot2 with a cut-off of fold change > 2 and *p*-value < 0.05.

### Drug Repurposing Using the LINCS Drug-perturbation Data

DEGs from PCR arrays were first sorted by log2FC values. The up-regulated and down-regulated genes were then chosen to identify drugs and compounds against the LINCS database using the Connectivity Map Linked User Environment (CLUE) platform 42. The drug connectivity score (CS) with a negative value smaller than -80 was used to identify candidates and compounds.

### Cell Culture

Murine macrophage RAW264.7 cells and Human colon epithelial carcinoma cell lines, SW480 (ATCC, CCL-228) and DLD-1 (ATCC, CCL-221) cells were maintained in RPMI 1640 (Gibco, #A1049101) and DMEM/F12 (Gibco, #11330032) medium, supplemented with 10% fetal bovine serum, 1X penicillin-streptomycin solution (Gibco, #15140163) at 37 °C with 5% CO_2_.

### Anti-inflammatory Assay

The cytotoxicity of brefeldin-a (BFA, MedChemExpress, HY-16592) and flubendazole (FLU, MedChemExpress, HY-B0294) on SW480 and DLD-1 cells was assessed by cell counting kit-8 (CCK-8) assay (APExBIO, #K1018). Cells were cultured overnight in 96-well (1 × 10^4^ cells/well) plate. Drugs were diluted in distilled water to reach final concentrations of 0.01, 0.1, 1, 10, and 25 µM and 0.05% DMSO as the negative control. Plates were cultured with the fresh drug-containing medium at 37 °C for 24 h. The absorbance of each well was determined by using a 96-well multiscanner. After subtracting background absorption, the results were expressed as a percentage of viability relative to control cultures. Drug concentrations that inhibited cell viability by 50% (IC_50_) were determined using GraphPad Prism 8 software.

RAW264.7 and SW480 cells were plated in 6-well (5 × 10^5^ cells/well) plates for ELISA or RNA/protein isolation, respectively. To evaluate the anti-inflammation efficacy of BFA and FLU, RAW264.7 cells were stimulated with *Escherichia coli*-derived lipopolysaccharides (LPS, O111:B4; SIGMA, L3024) at 50 ng/ml concentration for different times alone or with BFA at indicated concentrations in triplicates. The supernatant was collected for ELISA detection. SW480 cells were stimulated with LPS (50 ng/mL) alone or with BFA (25 µM) for 24 h. Total RNA was isolated from cells using TRIzol reagent and reverse transcribed to cDNA. Changes in gene transcripts of *CGASs*, *STING, IFN-β,* and* IFIT-1* were analyzed by RT-qPCR. The total protein lysates were collected from SW480 cells using RIPA buffer. Changes in protein levels of cGAS, STING, TBK1, NFκB, pro-IL-1β and pro-Casp-1, mature IL-1β (p17), cleaved Casp-1 (p20), and NLRP3 (from CST, Invitrogen, and Santa Cruz) were detected by western blot. Three technical replicates were used for each sample.

### Mouse Model Validation of Drug Efficacy

Acute colitis was induced in mice by 3% DSS in drinking water *ad libitum* for seven days and followed with four days of normal distilled water. Control mice were fed with distilled water for 11 days. The colitis group mice were randomly grouped and intraperitoneally injected with 100 µL vehicle (0.01% DMSO) or BFA (2.5, 5, and 10 mg/kg) at day 4 and 6 post-DSS. Mice were evaluated daily for changes of body weight. At day 9 and 11, mice were euthanized to harvest colons and blood for further analysis. Colon lengths were measured at autopsy. Mouse colon biopsies were lysed in HTAB buffer to determine the MPO activity and in RIPA buffer for IL-1β secretion (ELISA) and cGAS expression (immunoblotting). Serum was separated from clotted blood by centrifugation. Serum AST and ALT were measured by ELISA (IT5530 and IT5508, G-Biosciences) according to the manufacturer's instructions.

### Statistical Analysis

All data are mean ± s.d. For animal experiments, GraphPad Prism software version 8 (GraphPad Software, CA) was used for the preparation of graphs and comparison by using unpaired t-test, one-way ANOVA (Tukey's post hoc), or two-way ANOVA between groups, Kaplan-Meier curves (log-rank test) for survival analysis. Statistical analysis was also carried out in R (version 4.0.2) and plotted using ggplot (version 3.3.2) 21 and pheatmap (version 1.0.12) (https://CRAN.R-project.org/package=pheatmap). For all experiments, *P* < 0.05 was considered to be statistically significant.

## Results

### cGAS is Positively Associated with Human IBD

**Figure 1:**
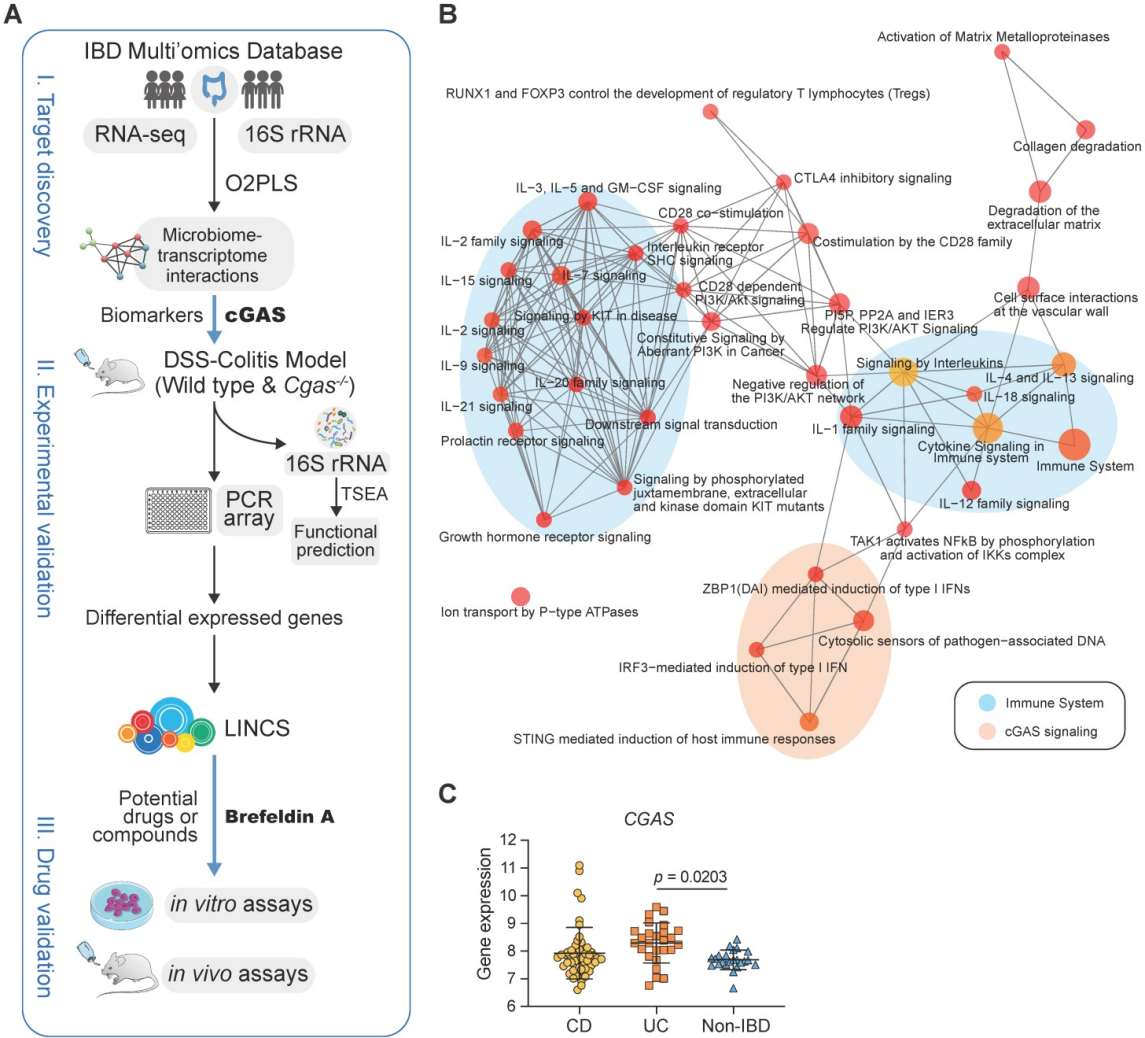
** cGAS is Highly Associated with Human IBD. (A)** Workflow for the exploration of drugs for IBD including three steps: I) target discovery, identify highly inter-correlated microbiome-transcriptome interactions by using O2PLS based on the human cohort to reveal potential biomarkers in IBD; II) experimental validation, studies on intestinal bacteria for functional prediction based on TSEA and transcriptomes for drug screening based on LINCS, respectively; III) drug validation, showing the working model of brefeldin-a for treatment of IBD. **(B)** Functional enrichment network of Reactome based on the top 200 highly inter-correlated genes. cGAS signaling pathway and downstream effectors are highlighted in orange. Each node corresponds to a Reactome term; the node's size is the number of genes annotated to the term. Edges between nodes indicate genes shared between Reactome terms. IL, interleukin. **(C)** cGAS expression levels in the rectum. n = 49, 26, 23 independent samples for CD, UC, non-IBD. One-way ANOVA with Tukey's post hoc test determined significance. See also Figure S1.

To identify the potential pathways in the pathogenesis of IBD, we built a novel potential strategy based on host-microbiome interaction for biomarker discovery and drug repurposing for IBD (Figure 1A). We analyzed the host transcriptome data from the publicly available dataset (IBDMDB), including 90 participants (43 CD, 25 UC, and 22 non-IBDs (controls), with no statistically significant differences in age and BMI (Figure S1A, Table S1). A total of 243 biopsies of transcriptional profiling were selected, including 98 rectal, 53 colonic, and 92 ileal subjects. We observed a clearer separation between ileum with colon or rectum samples (Figure S1A), consistent with the previous analysis that tissue location was a driver of intestinal gene expression 10.

To identify genes that are highly related to gut microflora in IBD, a total of 4,811 differentially expressed genes and 169 bacteria were selected to identify the joint covariation between the transcriptomic and microbiome datasets by using the O2PLS approach and the loading value were shown in Table S2. The joint variation between UC and non-IBD subjects revealed a strong positive linear correlation (R^2^ = 0.711; Figure S1B). Reactome pathways 24 enrichment analysis confirmed that the immune-related pathways were significantly over-represented (Figure 1B, Table S3). In particular, cGAS signaling and its downstream effectors were significantly enriched, including cytosolic sensors of pathogen-associated DNA, STING-mediated induction of host immune responses, and IRF3- or ZBP1(DAI)-mediated induction of type I IFN pathways.

The cGAS-STING pathway was enriched in up-regulated genes in ileum biopsies with CD and in rectum with both CD and UC, which had the same trends as IL-17 signaling pathway 43 and the complement cascade 10 that have been confirmed by overrepresentation enrichment and implicated in the inflamed intestines of IBD (Figure S1C). Compared to subjects without IBD, *cGAS*, *STING,* and* TNF* involved in the cGAS-STING signaling and *IL1B* and *MUC5AC* involved in IL-17 signaling pathways were significantly differentially expressed in inflamed rectum biopsies (Figure 1C, S1D). Overall, these data supported that cGAS may play a causal role in colitis.

### Colitis and Dysbacteriosis Activate cGAS and Induce Type I IFN in Mice

We next used a standard model of 3% DSS (epithelial irritant) in drinking water *ad libitum* to induce IBD (Figure S2A). This colitis model was appropriately established by DSS treatment. It significantly aggravated colitis's clinical signs, including weight loss, DAI, colon length, colonic MPO activity, pathological changes, and goblet cell secretion (Figure S2B-E, S2J-K). On the 9^th^-day post DSS challenge, where the severity of colitis nearly reached its peak, we observed a dramatic up-regulation of cGAS and downstream effectors at protein and mRNA levels in the inflamed colon following DSS treatment compared to the drinking water groups as controls (Figure S2F-G). Higher levels of type I IFN induction IL-1β and TNFα were observed in the colon of DSS-exposed mice (Figure S2H-I). Taken together, these findings indicate that cGAS may play a vital role in promoting innate immune responses in IBD.

Next, we used mouse models of antibiotic-associated diarrhea to investigate the effects of the dysbiosis of the gut microbiota on cGAS signaling. Mice were treated with antibiotics cocktail (MIX group), including streptomycin (STR), vancomycin (VAN), colistin (COL), and ampicillin (AAM), or treated individual antibiotic in the drinking water *ad libitum* (Figure S3A). The MIX mice with acquired depleted intestinal microbiota were usually considered as pseudo-germ-free. STR, VAN, and AAM treatment regimens caused a higher degree of colonic shortening, while MIX mice showed no colonic changes (Figure S3B). Histopathological assays showed no structural changes of the colon in the MIX mice, while the STR, VAN, COL, and AAM groups displayed a varying degree of pathological changes including inflammatory infiltrates, destroyed crypts, and goblet cell loss (Figure S3E-F). cGAS pathway activation was shown by overexpression in STR, VAN, and COL mice, although the degree was different (Figure S3C-D). Our data showed that dysbacteriosis-induced intestinal inflammation resulted in cGAS activation. The differences observed between MIX and single antibiotic-treated mice may be explained by the absence of the normal microbiota or the presence of a remnant microbiota 44.

### Loss of *Cgas* Attenuated Inflammatory Responses

**Figure 2:**
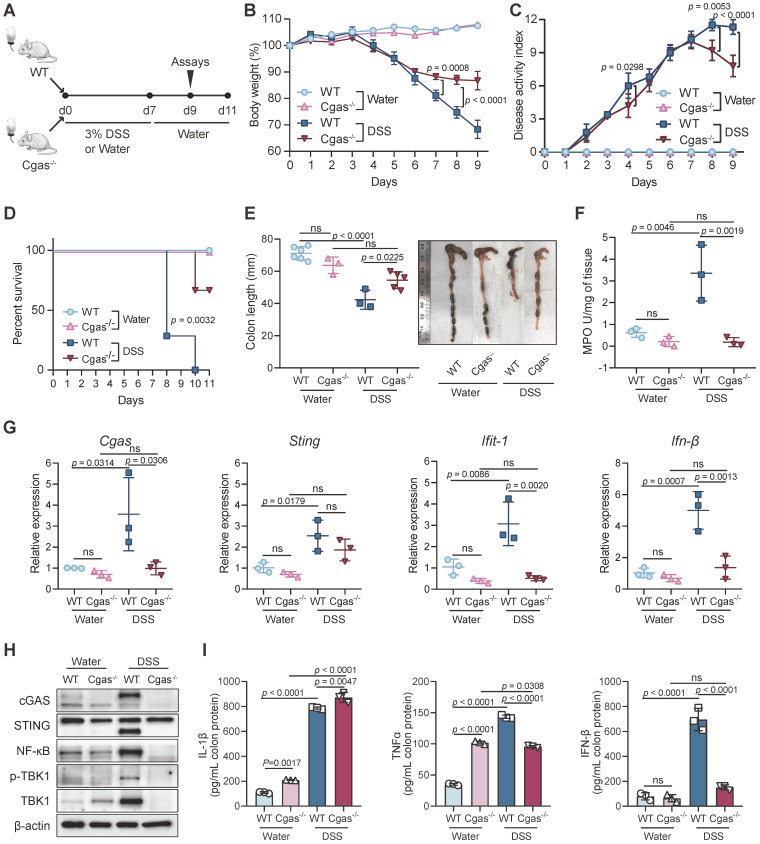
** cGAS Regulates Acute Intestinal Inflammation. (A)** Experimental design. WT and *Cgas*^-/-^ mice treated with water or 3% DSS in drinking water for 7 days and followed for normal water for 4 days. **(B)** Weight loss in DSS-exposed mice. **(C)** Disease activity index (DAI) of DSS-exposed mice. **(D)** Kaplan-Meier survival curve for 11 days after DSS treatment. **(E)** Length of the colons of WT or *Cgas*^-/-^ mice treated with DSS or control at day 9. **(F)** Myeloperoxidase (MPO) activity in colon homogenates at day 9. **(G)** Colonic *Cgas*, *Sting, Ifit-1,* and *Ifn-β* transcripts determined in WT or *Cgas*^-/-^ mice undergoing DSS-colitis at day 9. **(H)** Western blot analyses of colons at day 9. Lysates were probed against cGAS, STING, p65, p-TBK1, TBK1, and β-actin. **(I)** Inflammatory cytokine expression was quantified in whole colon tissues from WT or *Cgas*^-/-^ mice at day 9 by ELISA. Data are presented as the mean ± s.d. n = 5-7 mice/group from three independent experiments; statistical significance determined by one-way ANOVA (Turkey's post hoc) or two-way ANOVA. *ns*, no significant. See also Figure S4.

To explore the effect of cGAS loss on the colon, we performed DSS-colitis experiments in cGAS germline knockout (KO) mice (Figure 2A). The *Cgas*^-/-^ mice have no apparent gross phenotype and no aberrant colonic inflammation compared with WT control mice (Figure 2B-D). No significant difference was found in intestinal shortening, MPO activity, and injury or proliferation between WT and cGAS KO mice (Figure 2E-F, S4A-C). MUC5AC is a major gel-forming mucin secreted by goblet cells, which has shown a protective role against helminth infection in mice 45 and controls injury in the acutely inflamed colon 46. The number of goblet cells and the fluorescence intensity of *Muc5ac* in intestines were not significantly altered in KO mice than in the controls (Figure S4D-E). Immunoblotting of colon extracts confirmed the absence of cGAS (Figure 2H).

After 7 days of DSS treatment following 4 days of recovery, *Cgas*^-/-^ mice exhibited dramatically reduced disease severity as measured by less weight loss, DAI scores, colonic shortening, and MPO expression (Figure 2B-C, 2E-F). WT mice began to die after 8 days of DSS induction. No *Cgas*^-/-^ mice died within 9 days, which indicates a higher survival rate compared to WT mice (Figure 2D). Furthermore, *Cgas*^-/-^ mice presented significantly decreased intestinal injury and inflammation to those in WT mice, displaying only mild histological inflammation features, less inflammatory infiltration, and no gross ulceration or overall tissue damage in colons and small intestines at day 9 (Figure S4A-C). Consistent with the reduced disease symptoms and inflammation,* Cgas*^-/-^ mice showed no decrease in goblet cell numbers and fluorescence intensity of Muc5ac in intestines by day 9 (Figure S4D-E). Importantly, inhibition of NF-κB, p-TBK1, and TBK1 at protein levels and transcription of *Ifit-1*, and *Ifn-β* were observed in the colon of cGAS deficient DSS-colitis mice (Figure 2G-H). The colonic pro-inflammatory cytokines TNF-α and IFN-β were also decreased, while IL-1β increased in *Cgas*^-/-^ mice compared to WT mice (Figure 2I). Taken together, our results demonstrated that cGAS plays an essential role in promoting the inflammatory response in colitis.

### cGAS Deficiency Alters the Composition of Gut Microbiota

**Figure 3:**
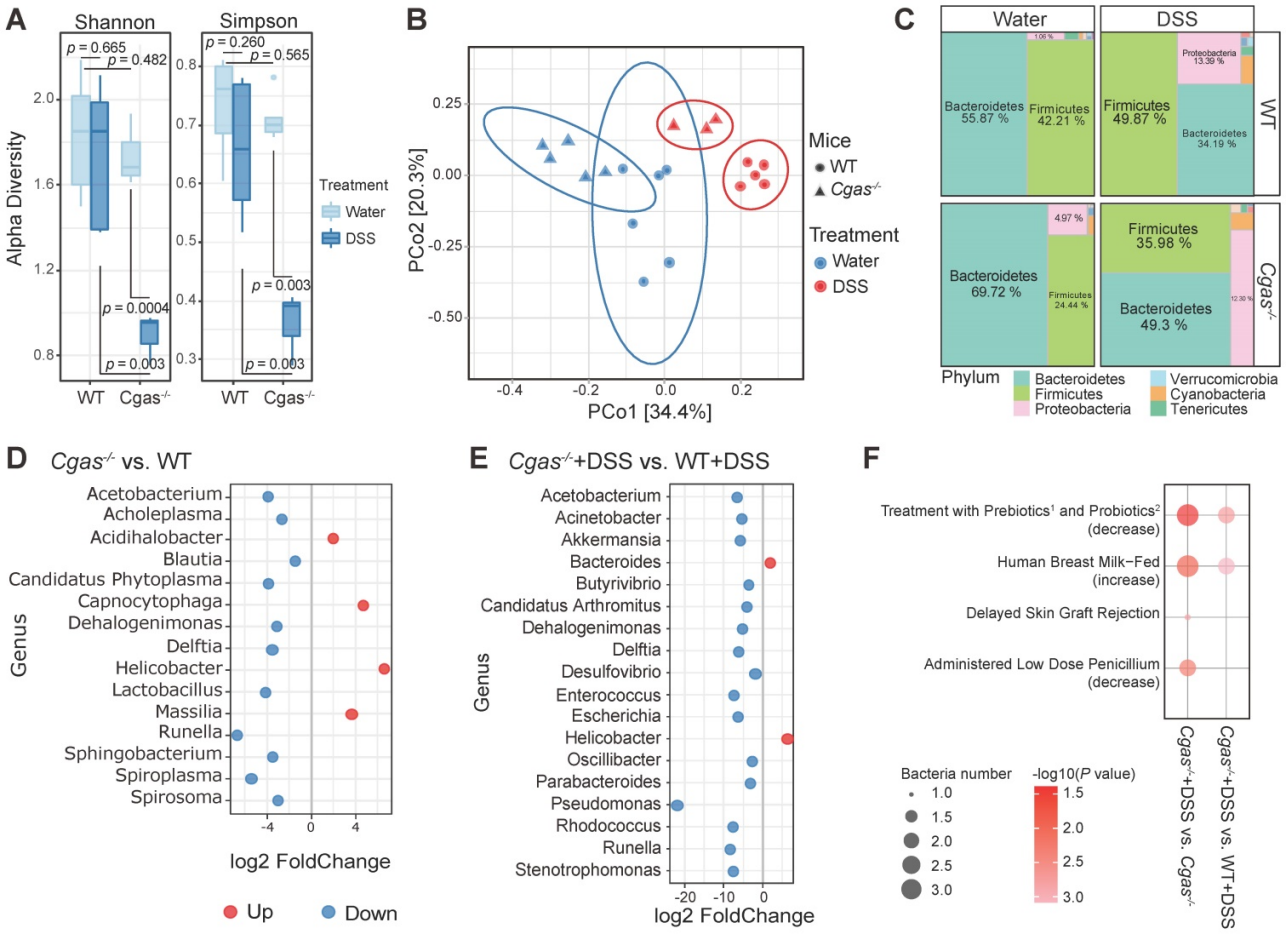
** Difference of Microbiota in Stool Samples between WT and *Cgas*^-/-^ Group. (A)** Alpha diversity metrics to measure the richness of the communities in the feces. Dissecting filtered bacteria with Shannon and Simpson indexes (significance analyzed by t-test). Colors stand for different treatments. **(B)** Principal Coordinates Analysis (PCoA) based on bacterial 16S rRNA gene sequence abundance in fecal content. All samples were subjected to the beta diversity analysis of bacteria abundances generated using the Bray-Curtis distance metric. The shape of the dot represents different groups. Colors stand for different treatments. **(C)** Bacterial-taxon-based analysis at the phylum level in the feces. Relative abundance between different groups was expressed as a percentage (%) represented as treemap layout. Colors indicate the phylum information. Ten most abundant phyla of each group are shown. **(D-E)** Comparison of the significant differential microbiome at the genus level. Only bacteria with significant differences (*p-value < 0.05 & |log2Fold Change|>1*) between the WT and *Cgas*^-/-^ controls **(D)** and subjected to DSS **(E)** are depicted. **(F)** Taxon set enrichment analysis (TSEA) of potential biological taxon sets in inflamed *Cgas*^-/-^ versus control and WT. 1, GOS plus FOS, inulin and anthocyanins; 2, *Bifidobacterium lactis* HN019 and *Lactobacillus acidophilus* NCFM. Throughout, n = 6 in WT mice and n = 5 in cGAS KO mice for control, and n = 6 in WT mice and n = 3 in cGAS KO mice for DSS treatment. See also Figure S5-6.

To probe the impact of cGAS signaling on microflora, we explored the effect of cGAS deficiency on fecal microbiota with or without induction of acute colitis with DSS. No difference was found in observed microbial richness in WT mice between both phenotypes (Figure 3A). Moreover, *Cgas*^-/-^ mice showed no significant microbial richness difference from WT mice. On the other hand, DSS-treated *Cgas*^-/-^ mice showed significantly lower taxa richness than the *Cgas*^-/-^ control group and DSS-treated WT group. We discovered a distinct clustering of global microbiota composition between groups at the genus level driven by both phenotype (water vs. DSS, Pr(>F)=0.001) and genotype (WT vs. *Cgas*^-/-^, Pr(>F)=0.001) evaluated by PCoA analysis using Bray-Curtis distances metrics (Figure 3B). DSS treatment and *Cgas* deficiency strongly influenced the microbiota composition at the phylum level (Figure 3C, S5A). The most abundant phyla in WT vs. *Cgas*^-/-^ mice included *Bacteroidetes, Firmicutes*, and *Proteobacteria*. DSS-treated mice showed increased relative abundance of *Firmicutes, Proteobacteria*, and *Cyanobacteria*, and decreased relative abundances of *Bacteroidetes* vs. control mice.

To understand the phylogenic detail, we examined differential bacterial levels down to the genera and species classification (Figure 3D-E, S5B-C and S6, Table S4-5). Consistent with studies of microbiome in IBD patients, *Desulfovibrio, Enterococcus*, and *Escherichia* at the genus level, and *Desulfovibrio fairfieldensis, Escherichia coli*, and *Salmonella enterica* at the species level were more abundant, while genus *Prevotella* was significantly less prevalent in DSS-treated WT mice (Figure S5B, S6B). In the fecal pellets of *Cgas*^-/-^ control mice, most bacteria were significantly decreased vs. the WT control group, with genus *Helicobacter* and species *Bacteroides vulgatus* increased and genera *Blautia* and *Lactobacillus* decreased (Figure 3D, S6A). Significantly, after disease onset, the genera *Bacteroides* and *Helicobacter* were increased, and *Parabacteroides* and *Pseudomonas* were decreased in *Cgas*^-/-^ control and WT+DSS groups (Figure 3E, S5C and S6C-D). Strikingly, the genera *Desulfovibrio, Enterococcus*, and *Escherichia* were diminished in *Cgas*^-/-^ mice compared to WT mice under DSS treatment (Figure 3E). Meanwhile, *Bacteroides vulgatus* increased, and *Clostridium perfringens, Enterococcus faecalis, Escherichia sp.*, and *Salmonella sp*. decreased at the species level (Figure S6D).

To determine intrinsic and extrinsic factors associated with the disruption on microbial communities, we performed taxon set enrichment analysis (TSEA) among the microbiome differentially between inflamed *Cgas*^-/-^ versus control and WT+DSS groups (Figure 3F, Table S6). Interestingly, the cGAS-associated microbiome was predicted affecting pathways including treatment with prebiotics (GOS plus FOS, inulin and anthocyanins) 47 and probiotics (*Bifidobacterium lactis HN019* and *Lactobacillus acidophilus NCFM*) 48, human breast milk-fed 49, and administered low dose penicillium 50, which have been implicated to be beneficial to IBD or irritable bowel syndrome. In summary, these observations indicate that cGAS has a functional role in shaping the bacterial gut microbiota and is required to adjust microbiota expansion in colitis, which would be a niche of host-microbiome based potential therapeutic strategy.

### Loss of *Cgas* Affects IBD-Associated Genes Expression

**Figure 4:**
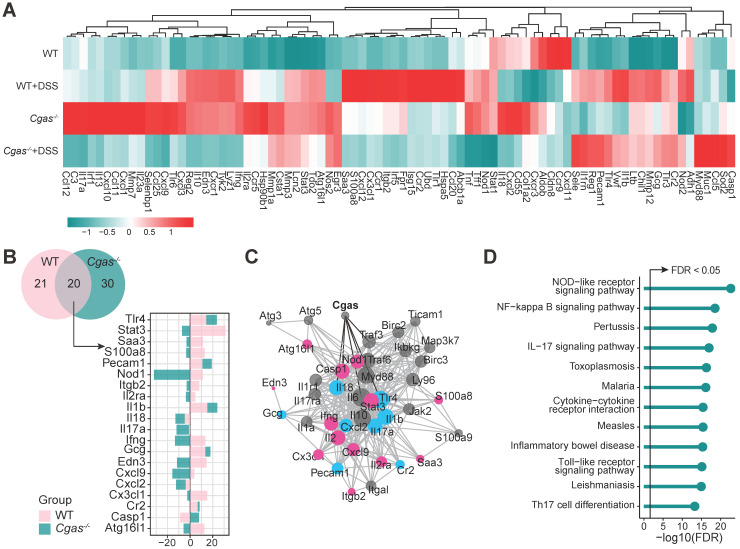
** Real-time PCR-array Analysis of Dysregulated Genes Implicated by cGAS Gene Depletion.** PCR-array analysis of colon tissue from WT and *Cgas^-/-^* mice subjected to DSS-induced colitis at day 9 (n = 5 per genotype for control and DSS treatment groups).** (A)** Heatmap represents up- and down-regulated genes profiling among groups. Genes with higher expression are depicted in red, with lower expression in green. **(B)** Venn diagram represents the number of genes significant DEGs between WT and *Cgas^-/-^* groups subjected to DSS-induced colitis (*p* < 0.05). The barplot shows the log2 fold change of the shared significant DEGs. Colors stand for different groups. **(C)** Protein-protein interaction network built using STRING. The nodes represent the proteins (genes); the edges represent the interaction of proteins (genes); blue and pink represent the same or opposite direction of gene changes in WT and *Cgas^-/-^* groups, respectively.** (D)** Lollipop chart represents KEGG pathways associated genes in Figure 4C displayed as the -log10 (FDR). The line is drawn at FDR = 0.05. FDR, false discovery rate.

To explore the effect of cGAS on the gene expression relevant to mouse IBD, we compared the expression of 85 genes in colons, including inflammation, immunity, apoptosis, cell adhesion, and known IBD-related genes, in WT and *Cgas*^-/-^ mice before and after DSS-colitis on day 9 (Figure 4A, Tables S7 and S8). A total of 20 differentially expressed genes were detected both in WT and *Cgas*^-/-^ mice, including *Tlr4* and *Il18* undergoing the same change, and *Stat3, Ifng, Cxcl9, Atg16l1*, and *Casp1* changing in the opposite direction (Figure 4B). Furthermore, we mapped these shared DEGs between WT and* Cgas*^-/-^ mice based on the STRING database to obtain potentially linked genes (Figure 4C), and the Mb21d1(CGAS) gene showed association with *Tlr4, Il18,* and* Casp1*. Enriched pathways from STRING showed that the induced transcripts may be involved in inflammatory bowel disease and dominance of immune-related pathways, including NF-kappa B, IL-17, Toll-like receptor signaling pathways, and Th17 cell differentiation, confirming the specific role of cGAS and its potential interactions in response to bacterial infections under the colitis (Figure 4D).

### Prediction of Candidate Compounds for IBD

**Figure 5:**
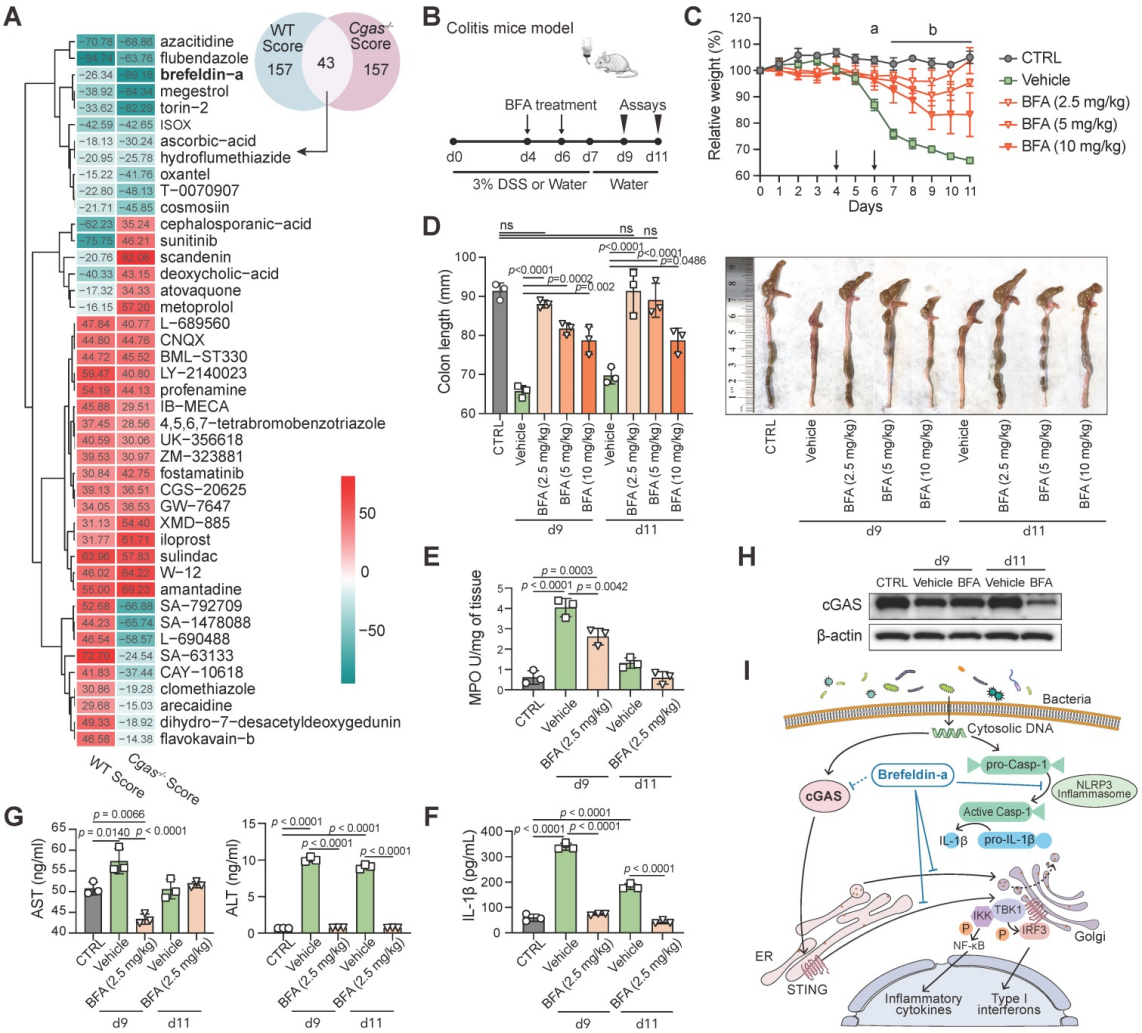
** Drug Repurposing on the LINCS Database for IBD. (A)** The Venn diagram represents the top 200 drugs using the DEGs from WT and *Cgas^-/-^* groups under DSS and water treatment searching against LINCS, individually. Heatmap shows the common 43 candidate drugs with the score values. **(B)** Experimental design. B6 WT mice were treated with water or 3% DSS in drinking water for 7 days and followed with drinking water for 4 days. Brefeldin-a (BFA, 2.5, 5, and 10 mg/kg) or vehicle (0.01% DMSO) was injected intraperitoneally (i.p.) in mice at day 4 and day 6 post-DSS. **(C)** Weight change. a, *p* < 0.0001, 0.0001 and 0.0004 (2.5, 5, and 10 mg/kg BFA groups) for comparison with vehicle group at day 6 post-DSS; b, *p* < 0.0001 for comparison with vehicle group. **(D)** Lengths and gross morphological changes of colons. **(E)** Colonic MPO activity. **(F)** Colonic IL-1β levels. **(G)** Serum AST and ALT levels were quantified by ELISA. **(H)** Western blot measured cGAS expression in the colons of these mice. **(I)** Schematic diagram of BFA exerting anti-inflammatory activity through inhibiting cGAS-STING pathway. In addition to blocking the protein trafficking from ER to the Golgi and the translocation of STING to the ER-Golgi intermediate compartment, BFA inhibits cGAS to block type I interferon production. BFA also effectively impairs NLRP3 inflammasome activation. Data are presented as the mean ± s.d. by two-way ANOVA or one-way ANOVA (Turkey's post hoc); n = 5 mice per group from three independent experiments. See also Figure S7.

To identify potential drugs and compounds that can be used for IBD treatment, we first selected the over- and under-expressed genes between control and DSS treatment groups in WT and *Cgas*^-/-^ mice as a disease signature, and then used these signatures to query in CLUE platform, respectively. The closer the CS is to -100, the better the response showed by drugs to reverse the expression of DEGs. The top 200 up- and down-scored medications were picked out with the lowest CS score, in which 43 common candidate drugs were in WT and *Cgas^-/-^* groups under DSS and water treatment at day 9 (Figure 5A, Table S9). 7 of the 43 candidates were published relevant to IBD. Azacitidine (DNA methyltransferase inhibitor) 51, ISOX (HDAC inhibitor) 52, ascorbic-acid (antioxidant) 53, cosmosiin (cytochrome P450 inhibitor) 54, IB-MECA (adenosine receptor agonist) 55, fostamatinib (SYK inhibitor) 56, and iloprost (platelet aggregation inhibitor) 57 have been used as a treatment or providing protective effects for IBD.

Among the top compounds, we investigated the anti-inflammatory activity of the protein synthesis inhibitor brefeldin-a (BFA) and the tubulin inhibitor flubendazole (FLU), which have not been previously studied in IBD. The anti-inflammatory activities of these drugs were examined *in vitro* in LPS-induced murine macrophage RAW264.7 cells. BFA decreased LPS-induced IL-6 and TNFα in the supernatant of RAW264.7 in a dose-dependent manner; however, FLU increased IL-6 at high concentrations (Figure S7A). Therefore, we next focused on the role of BFA, whose treatment also inhibited IL-1β, IL-6, TNFα, and IFN-β in a time-dependent manner compared to vehicle controls (Figure S7D). BFA showed less cytotoxicity in SW480 human colon cancer cell line (IC50=4.652 µM) than that in DLD-1 cells (IC50=0.167 µM) (Figure S7B-C). Thus, SW480 cells were selected for subsequent experiments. To validate the mechanism of drug therapy, we observed decreased transcript and protein levels of the cGAS-STING pathway and NLRP3 inflammasome in LPS-induced SW480 by qPCR and western blot (Figure S7E-F).

The therapeutic application of BFA was also examined in the acute 3% DSS-induced colitis model (Figure 5B). BFA-administered mice were protected from DSS-induced inflammatory disease with less weight loss (Figure 5C), DSS-induced colon shortening (Figure 5D), reduced MPO activity (Figure 5E), and decreased colonic inflammatory cytokine IL-1β (Figure 5F). We next evaluated the impact of BFA doses on the preventive efficacy. Remarkably, the low concentration of BFA (2.5 mg/kg) produced a more pronounced effect than higher concentrations (5 and 10 mg/kg). Furthermore, BFA had no effect on the serum AST and ALT levels in both DSS treatment and vehicle groups, indicating that it has not caused liver toxicity (Figure 5G). Consistent with the above cell experiment, BFA treatment can inhibit the expression of cGAS in the colon (Figure 5H). These results suggest that BFA can inhibit inflammatory response and mitigate colonic inflammation, through blocking cGAS (Figure 5I), which may be exerted by inhibiting NLRP3 inflammasome 58 and STING 59. Taken together, we identified a top drug candidate BFA for potential IBD treatment consideration through the drug repurposing approach and validated the anti-inflammatory activity and potential mechanisms *in vitro* and *in vivo*.

## Discussion

In this study, we designed a novel potential strategy based on host-microbiome interaction for biomarker discovery and drug repurposing for IBD (Figure 1A). By integrating public transcriptomic and microbiome data sets from IBD cohorts, we observed a significant correlation between cGAS signaling and IBD-associated genes and pathways. Hence, we focused on cGAS and revealed increased cGAS in IBD patient biopsies and experimental models, which subsequently activated immune reactions and inflammatory responses that may be associated with gut microbiota.

Previous studies have generally focused on the function of the cGAS-STING pathway in viral infection, cancer immunity, and autoimmune disorders 12. Our results demonstrated that cGAS was significantly up-regulated in inflamed human samples with CD and UC, and also in two independent experimental models, DSS-colitis and antibiotic-associated diarrhea. Critically, we analyzed transcriptome and microbiome data of IBD patients and screened out cGAS as a potential biomarker and drug target. Further analysis using genetic knockout of cGAS in the inflammatory model strongly supported our notion that cGAS inhibition could rescue aspects of the inflammatory phenotype, consistent with the literature in other autoimmune disorders 60. In IBD, ATG16L1 mutations promote IL-22 production through the STING pathway to excess epithelial cell death 13, while clear evidence for cGAS' role in IBD has not been presented. Recent research has implicated that STING agonists might have a potential therapeutic benefit in anticancer immune therapy 61 and antiviral responses 62, and STING inhibition may be promising for treating inflammatory diseases (such as AGS) through genetic approaches 63 or chemical inhibitors 64. Accordingly, therapeutic avenue through targeting the cGAS-STING pathway may be realized one day for infections, autoimmunity, inflammatory diseases and cancers.

We subsequently investigated variations in the composition and relationship of host colonic transcriptomes and the intestinal microbiota in humans and mouse models. First, we found that cGAS was critically associated with the abundance of specific microbial taxa. cGAS-associated changes impacted multiple gut microbiota, such as *Bacteroides*, *Lactobacillus, Prevotella,* and *Pseudomonas*. Some of them are generally associated with gut health, including *Bacteroides*, *Bifidobacterium,* and *Prevotella* as key SCFA producers 65, 66, *Coprococcus* and* Lachnospiraceae* for producing butyrate 65, 67,* Blautia* relating to bile metabolism 65, 68, and *Lactobacillus* exhibiting probiotic ability 68. On the other hand, *Desulfovibrio* 69*, Enterococcus* 70, *Escherichia* 71*,* and *Pseudomonas* 72 are frequently found at high ratios in IBD patients than healthy individuals and considered to be potential pathogens. Interestingly, *Helicobacter* has been recognized as a primary pathogenic factor for gastric and duodenal ulcers and gastric adenocarcinoma but prevents allergic disorders and chronic inflammatory conditions (i.e., IBD) in increasingly robust experimental and epidemiological evidence 73. Additionally, we observed significantly increased strain diversity in beneficial species such as *Anaerostipes hadrus* 74, *Bacteroides vulgatus* 68*,* and* Faecalibacterium prausnitzii* 75 in cGAS deficient mice, which may play unknown roles in inflammation-associated immunosuppression.

Notably, our functional enrichment analysis predicted several potential targets to develop effective therapies. By using TSEA 40 analysis, our microbiome data were functionally profiled and compared to public datasets and known microbial signatures. *Acinetobacter, Alistipes*, and *Parabacteroides* (decreased abundance in *Cgas*^-/-^+DSS group) were enriched in prebiotics treatment (GOS plus FOS, inulin and anthocyanins), which significantly decreased the pro-inflammatory cytokines both in infected Caco-2 cells and post-infectious irritable bowel syndrome mouse models 47, and probiotics treatment (*Bifidobacterium lactis* HN019 and *Lactobacillus acidophilus* NCFM), which has positive effects on the treatment and maintenance of UC and other associated IBD pathologies (pouchitis and cholangitis) 48. *Acinetobacter, Klebsiella*, and *Pseudomonas* (decreased abundance in *Cgas*^-/-^+DSS group) were enriched in human breast milk-fed, which is a protective factor against aberrant intestinal inflammation and necrotizing enterocolitis in preterm infants 49. *Candidatus Phytoplasma* and *Prevotella* (decreased abundance in *Cgas*^-/-^+DSS group) were enriched in administered low dose penicillium, which reduces Th17 cells and subsequently suppresses the mouse colitis induced by DSS 50.

In addition to the functional prediction from the microbiome, we took a drug repurposing approach to identify drugs with potential to treat IBD using CLUE with the significant DEGs between cGAS KO and WT after DSS treatment. Forty-three common candidate drugs were identified in both WT and *Cgas^-/-^* groups, which can reverse the DEGs in inflamed colons Remarkably, seven candidate compounds in our screened results have already been reported for their potential protective or therapeutic effects in IBD 51-57. The DNA methyltransferase inhibitor azacytidine has been indicated to induce complete remission in myelodysplastic syndrome associated with IBD by suppressing pro-inflammatory cytokine responses 51. There were no reports on the direct association between ISOX and IBD, but some other HDAC inhibitors are already tested as an anticancer treatment, and one of them (SCFA butyrate) is used to treat IBD 52. Ascorbic acid (AA), known as vitamin C, is important for patients suffering IBD since IBD patients are at risk of vitamin C deficiency. AA reduced oxidative stress and inflammatory response in DSS-induced colitis and could modify the gut microbiota composition related to the pathogenesis of IBD 53. Since some critical CYP isoforms have been reportedly linked to metabolism and pathogenesis or etiology of UC, cosmosiin, as a cytochrome P450 (CYP) inhibitor, may play an effective role against IBD 54. The adenosine receptor agonist IB-MECA was shown to alleviate inflammation in DSS-induced colitis and spontaneous colitis found in IL-10-deficient mice 55. Fostamatinib, an SYK inhibitor, reduced the inflammatory damage in the acetic acid-induced colitis 56. Platelet aggregation inhibitor iloprost has been demonstrated to be an antioxidant and protective against colitis induced oxidative stress in experimental colitis 57. Hence our bioinformatics-based approach can effectively infer important repurposing drug candidates in both animal models and clinical trials.

To validate our drug repurposing findings, we chose to assess the anti-inflammatory properties of BFA and investigate a possible underlying mechanism of benefiting IBD patients. BFA has been widely studied for its antitumor, antifungal, and antiviral effects and apoptosis-inducing property 76. BFA not only effectively inhibits protein trafficking from the endoplasmic reticulum (ER) to the Golgi apparatus 58, but also blocks type I IFN production by impeding the translocation of STING to the ER-Golgi intermediate compartment 59. Apart from the above, we demonstrated that BFA inhibited inflammation through blocking cGAS expression and type I IFN response in colitis model and human colon cancer cells. Admittedly, acute DSS-induced colitis model may not accurately represent the IBD disease occurring in humans. Our validation assay presents a preliminary test for developing repurposing drugs, which is yet to be confirmed for its correlation with human IBD. Therefore, chronic colitis models and human specimens should be considered in future studies to clarify the significance of cGAS in the pathogenesis of human IBD as well as the efficacy and mechanisms of therapeutic candidates (i.e., BFA).

Altogether, we provide a new framework to identify biomarkers correlated with gut microbiota and immunity in the pathogenesis of IBD. We used these inflammation-correlated microbial and genetic variants as potential drug targets for IBD. Notably, our findings are twofold, including modified microbiota affecting the host's immune response and leading to intestinal inflammation, and in turn, the effect of the altered immune response in *Cgas*^-/-^ mice on the microbiota. Furthermore, we combined multi-pronged approaches for drug repurposing and validation, including bioinformatic analysis of IBD cohorts and drugs, gene transcriptome and protein interactome, as well as cell culture and animal experimental studies. Collectively, our studies here offered a promising systemic framework to connect disease gene expression with microbiome for biomarker and therapeutics discovery applicable to IBD and successfully demonstrated the potential therapeutic effects and underlying mechanisms of repurposing drugs. This strategy may be broadly applied to other cases and have the potential to speed up drug discovery.

## Supplementary Material

Supplementary figures.

Supplementary tables.

## Abbreviations

AAM: ampicillin
AGS: Aicardi-Gourtières syndrome
ALT: alanine aminotransferase
AST: asparate aminotransferase
BFA: brefeldin-a
Casp-1: caspase-1
CCK-8: cell counting kit-8
CD: Crohn's disease
cGAS: cyclic GMP-AMP synthase
CLUE: Connectivity Map Linked User Environment
COL: colistin
CS: connectivity score
CST: Cell Signaling Technology
DAI: disease activity index
DAPI: 4',6'-diamidino-2-phenylindole
DEGs: differential expressed genes
ELISA: enzyme-linked immunosorbent assay
ER: endoplasmic reticulum
FLU: flubendazole
GWAS: genome-wide association studies
H&E: hematoxylin and eosin
IACUC: Institutional Animal Care and Use Committee
IBD: inflammatory bowel disease
IBDMDB: IBD Multi's omics database
IC50: 50% inhibiting concentration
IFN: type I interferon
iHMP: Integrative Human Microbiome Project
IL: interleukins
i.p.: intraperitoneally
IRF3: interferon regulatory transcription factor 3
LINCS: Library of Integrated Network-Based Cellular Signatures
LPS: lipopolysaccharides
MPO: myeloperoxidase
O2PLS: orthogonal partial least squares
OCT: optimal cutting temperature
PAS: Periodic acid-Schiff
PCoA: Principal Coordinates Analysis
qPCR: Quantitative Polymerase Chain Reaction
RIPA: radiation immunoprecipitation
SLE: systemic lupus erythematosus
STING: stimulator of the interferon genes
STR: streptomycin
TBK1: TANK binding kinase 1
Tregs: regulatory T lymphocytes
TSEA: Taxon Set Enrichment Analysis
UC: ulcerative colitis
UND: university of North Dakota
VAN: vancomycin
WT: wild type
