# Supplementary material for: Microbial and genetic-based framework identifies drug targets in inflammatory bowel disease
Source: Theranostics. 2021 Jun 1;11(15):7491–506. doi: 10.7150/thno.59196 (PMC8210594; doi:10.7150/thno.59196)
Supplement: Supplementary file 1 — Supplementary figures. [file thnov11p7491s1.zip › Supplementary materials legends.pdf]

Figure S1. Related to Figure 1: cGAS signaling enriched in host-microbiome interactions in human IBD. (A) Overview of human cohort characteristics (90 participants with CD, UC, or non-IBD (control)). Principal components analysis (PCA) shows principal component 1 (PC1) and PC2 for RNA-seq data from ileal, colonic, and rectal biopsies from this study. **(B)** Scatterplot showing joint score vectors (transcript, microbiome) plotted against each other obtained from O2PLS. The coefficient of determination R^2 was 0.711. **(C)** Heatmaps show the expression of genes involved in cGAS-STING signaling, IL-17 signaling, and complement cascade from 243 subjects with ileal, colonic, and rectal biopsies. Ordering by diagnosis, clustering by enriched pathways. cGAS-STING pathway showed enrichment in ileum, colon, and rectum, which showed the same trends as IL-17 signaling and the complement cascade identified in IBD. **(D)** Expression of genes involved in the cGAS-STING pathway (*STING*, *TNF*) and IL-17 signaling (*IL1B, MUC5AC*) in the rectum. n = 49, 26, 23 independent samples of CD, UC, non-IBD. One-way ANOVA with Tukey's post hoc test determined significance.

Figure S2. Activated colonic cGAS-STING signaling pathway correlates with active inflammation during experimental DSS-colitis. (A) Experimental design. DSS (3% wt/vol) was administered in drinking water ad libitum to B6 WT mice. **(B)** Weight change of DSS-exposed mice. **(C)** Disease activity index (DAI) of DSS-exposed mice. **(D)** Length of the colons of mice treated with DSS and H_2O at day 9. **(E)** Colonic myeloperoxidase (MPO) activity. **(F)** Western blot analyses from the colon at day 9. Lysates were probed against cGAS, STING, p65, p-TBK1, TBK1, and β-actin. **(G)** Colonic *Cgas*, *Sting*, *Ifit-1*, and *Ifn-β* transcript expression was determined from mice undergoing DSS-colitis on day 9. **(H-I)** IL-1β **(H)** and TNFα **(I)** were quantified in the colon by ELISA. **(J-K)** Representative H&E **(J)** and PAS **(K)** stained images of proximal colon cross-sections on day 9 after initial DSS exposure; scale bar, 100 μm. Throughout, data are presented as the mean ± s.d. by two-way ANOVA or unpaired t-test. n = 5 mice per group from three independent experiments.

Figure S3. Clinical and histopathology data in antibiotic-treated mice. (A) Experimental design. Streptomycin (STR), vancomycin (VAN), colistin (COL), and ampicillin (AAM) alone or as a mixture of antibiotics (MIX) was administered in drinking water ad libitum to B6 WT mice. **(B)** Length of the colons of mice treated with antibiotics and H_2O on day 5. **(C)**

Immunoblot analysis determines the levels of cGAS, STING, p65, TBK1, TNF-α, and β-actin of the colon on day 5 after antibiotics treatment. **(D)** Transcript expression in the colon of antibiotic-treated mice. **(E-F)** Representative images of H&E **(E)** and PAS **(F)** stained proximal colon cross-sections on day 9 of DSS treatment (4% wt/vol); scale bar, 100 µm. In all panels, data are represented as mean ± s.d. **P < 0.01, ***P < 0.001, ****P < 0.0001, *ns* no significant. n = 4 mice per group from three independent experiments.

Figure S4. Related to Figure 2: Absence of cGAS prevents disruption of gut homeostasis. **(A)** Representative images of H&E-stained proximal colon (upper) and small intestine (lower) cross-sections at day 9 of treatment (3% wt/vol); scale bar, 100 µm. **(B-C)** Histopathology scores of colons **(B)** and small intestines **(C)** from DSS-colitis on day 9. n = 8 mice/group; statistical significance determined by one-way ANOVA with Tukey's post hoc test. **(D)** Representative micrographs of the proximal colon (upper) and small intestine (lower) PAS-stained cross-sections on day 9 after DSS-colitis; scale bar, 100 µm. **(E)** Double immunofluorescence for MUC5AC and DAPI in the colon (upper) and small intestine (lower) from WT and *Cgas*$^{-/-}$ mice before and after DSS treatments; scale bar, 20 µm.

Figure S5. Related to Figure 3: Comparison of significant differential bacteria at the genus level. (A) Relative abundance of fecal microbiota composition at the phylum level among groups (6 WT mice and 5 cGAS KO mice for control, 5 WT mice and 3 cGAS KO mice treated with DSS). Color indicates phylum information. **(B-C)** Comparison of the significant differential microbiome at the genus level. Only bacteria with significant differences (*p-value* < 0.05 *&* $|log2Fold\ Change| > 1$) between the WT+DSS and WT control groups **(B)**, *Cgas*$^{-/-}$+DSS and *Cgas*$^{-/-}$ groups **(C)** are depicted.

Figure S6. Related to Figure 3: Comparison of significant differential bacteria at the species level. (A-D) Comparison of the significant differential microbiome at the species level. Only bacteria with significant differences (*adjusted p-value* < 0.05 *&* $|log2Fold\ Change| > 1$)) between *Cgas*$^{-/-}$ and WT groups **(A)**, WT+DSS and WT control groups **(B)**, *Cgas*$^{-/-}$+DSS and *Cgas*$^{-/-}$ groups **(C)**, and *Cgas*$^{-/-}$+DSS and WT+DSS groups **(D)** are depicted.

Figure S7. Related to Figure 5: The anti-inflammatory effects of BFA and FLU. (A) IL-6 and TNFα levels in supernatants of RAW264.7 cells stimulated with LPS (50 ng/ml) with brefeldin-a (BFA) and flubendazole (FLU) (0.01, 0.1, 1, 10, 25 µM) for 24 h. 0.01% DMSO was used as vehicle control. **(B-C)** CCK-8 cytotoxicity assays were performed in SW480 **(B)** and DLD-1 **(C)** cells to BFA (0.01-50 µM). **(D)** IL-1β, IL-6, TNFα, and IFN-β in supernatants of RAW264.7 cells stimulated with LPS (50 ng/ml) and 25 µM BFA at different time points. 0.01% DMSO was used as vehicle control. **(E-F)** SW480 cells were stimulated with LPS (50 ng/ml) and treated with BFA (25 µM) or vehicle (0.01% DMSO) for 24 h. **(E)** *CGAS*, *STING, IFN-β,* and *IFIT-1* transcript expression in SW480 cells were determined by RT-qPCR. **(F)** Immunoblots determined protein expression of cGAS, STING, TBK1, NF-κB, pro-IL-1β and pro-Casp-1, mature IL-1β (p17), cleaved Casp-1 (p20), and NLRP3 in SW480 cells. Data are presented as the mean ± s.d. from three independent experiments.

Table S1. Characteristics of patients with IBD and non-IBD controls of the enrolled subjects.

Table S2. The loading values of genes and microbiomes for the joint covariance part.

Table S3. Reactome enrichment analysis results of the top 200 genes.

Table S4. List of differential microbiomes at the genus level. Between the WT and *Cgas*$^{-/-}$ controls (S4A) and subjected to DSS (S4B), WT+DSS and WT control groups (S4C), and *Cgas*$^{-/-}$ +DSS and *Cgas*$^{-/-}$ groups (S4D).

Table S5. List of differential microbiomes at the species level. Between the *Cgas*$^{-/-}$ and WT groups (S5A), WT+DSS and WT control groups (S5B), *Cgas*$^{-/-}$+DSS and *Cgas*$^{-/-}$ groups (S5C), and *Cgas*$^{-/-}$+DSS and WT+DSS groups (S5D).

Table S6. List of TSEA potential biologically taxon sets in inflamed *Cgas*$^{-/-}$ versus control and WT.

Table S7. Functional grouping and primers of the 85 genes included in the used PCR-array Mouse IBD.

Table S8. List of gene expression data of PCR arrays.

Table S9. List of potential drugs for treating IBD based on LINCS database and DEGs between WT and *Cgas*$^{-/-}$ mice treated with DSS. Connectivity scores were calculated from the CLUE platform. * The PMID numbers reference to the candidates published relevant to IBD.

Table S10. Source data.
