# Supplementary figures and images for: Microbial and genetic-based framework identifies drug targets in inflammatory bowel disease
Source: Theranostics. 2021 Jun 1;11(15):7491–506. doi: 10.7150/thno.59196 (PMC8210594; doi:10.7150/thno.59196)

**A**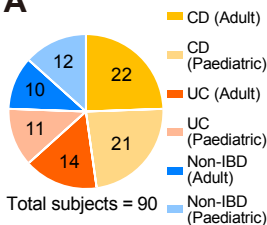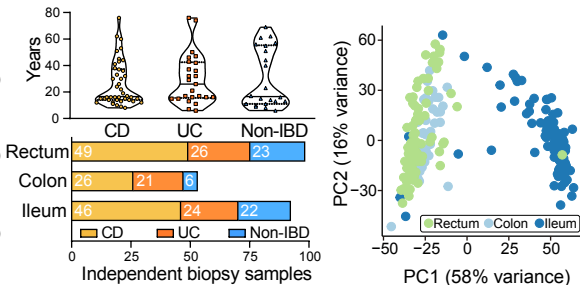**B**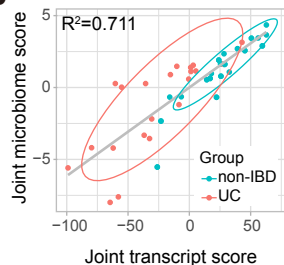**C**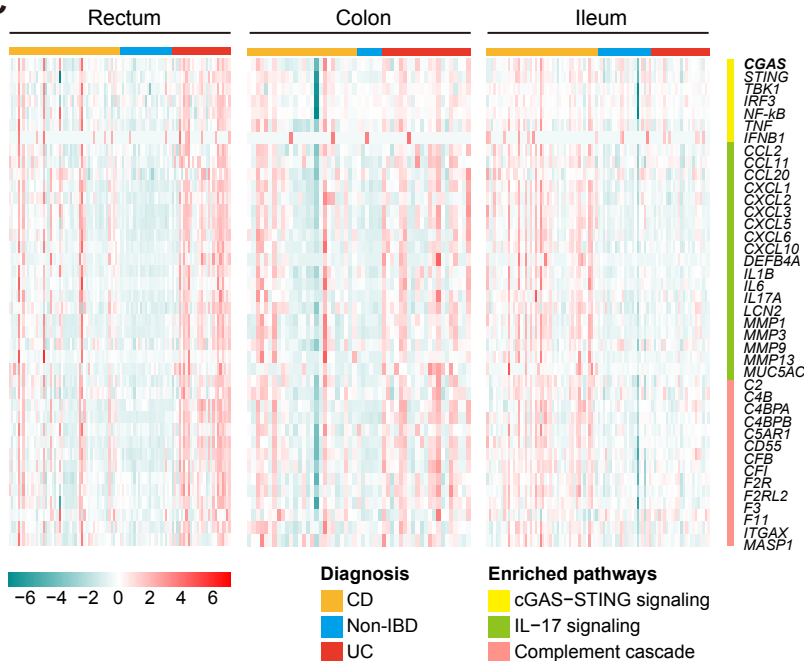**D**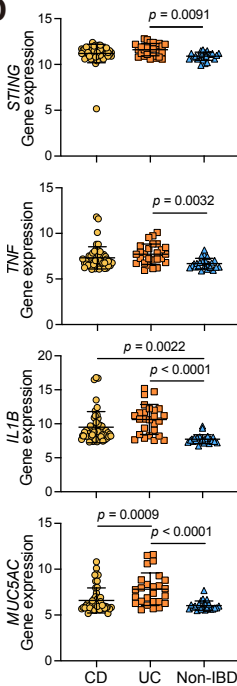

Supplement: Supplementary file 1 — Supplementary figures. [file thnov11p7491s1.zip › Figure S1.pdf]

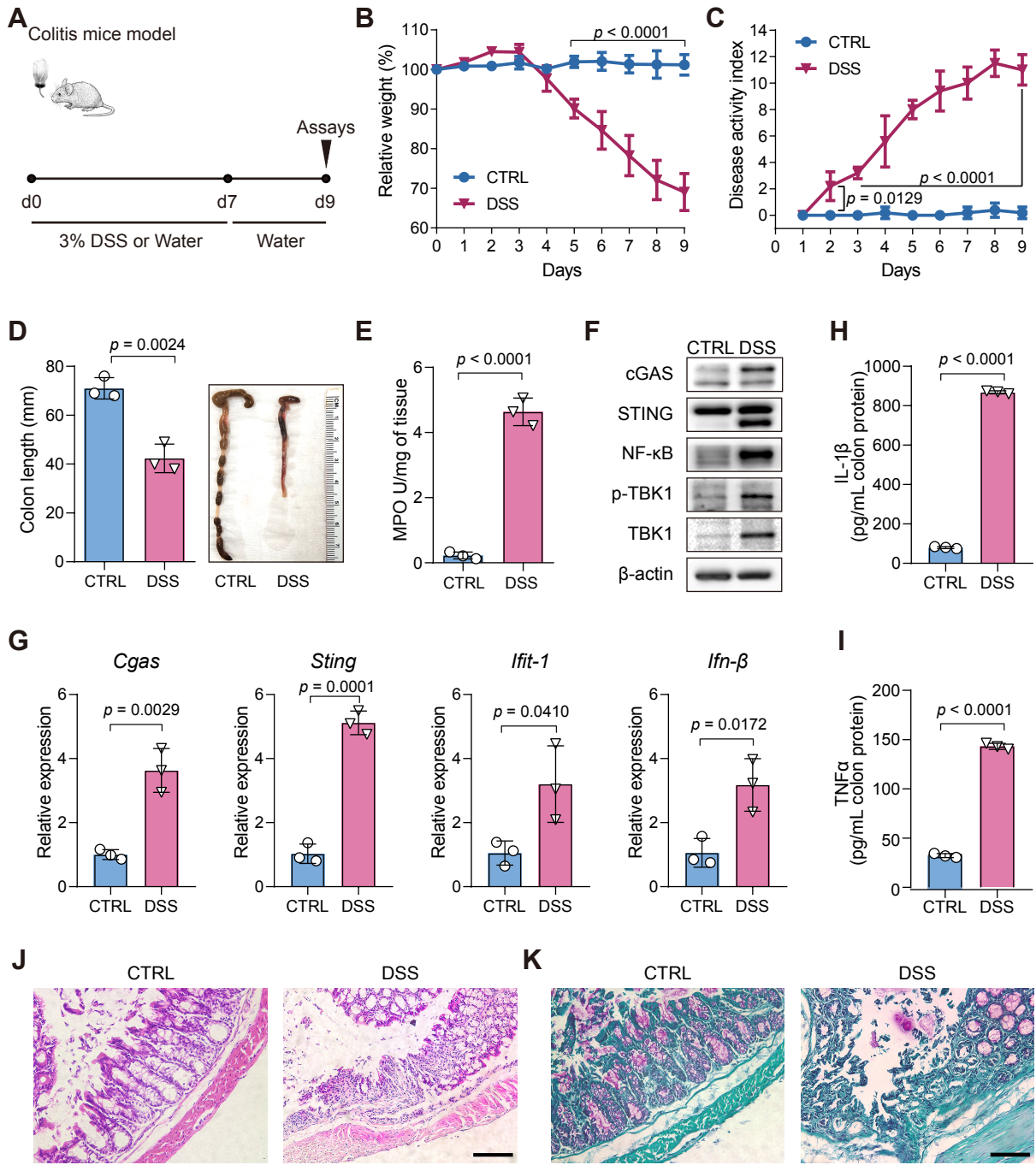

Supplement: Supplementary file 1 — Supplementary figures. [file thnov11p7491s1.zip › Figure S2.pdf]

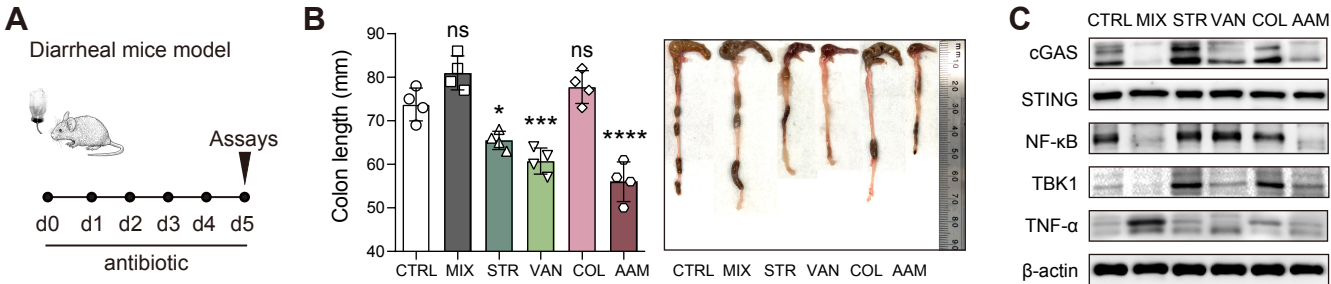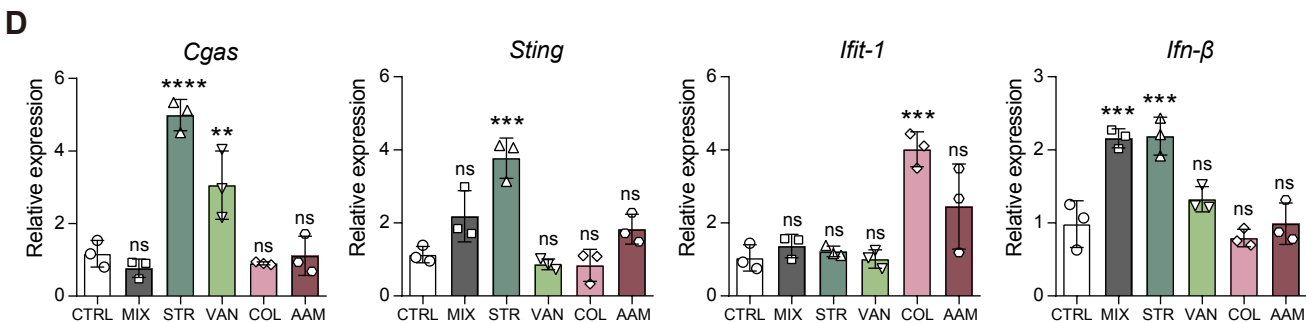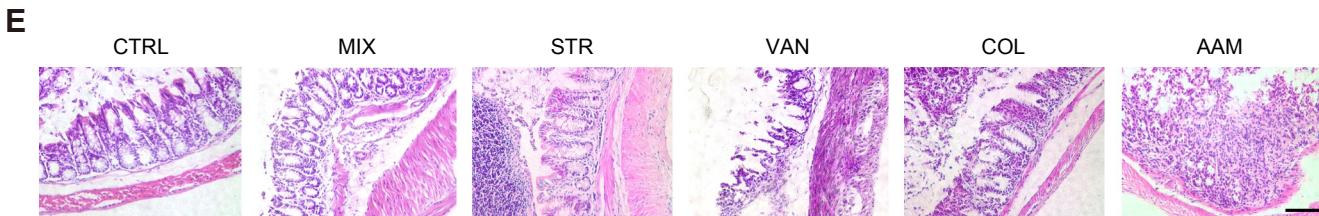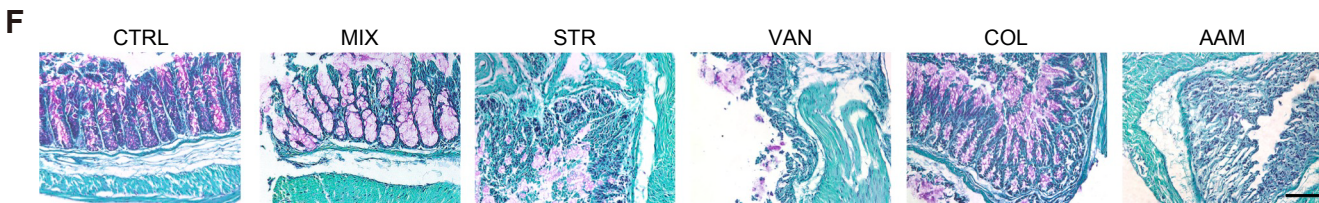

Supplement: Supplementary file 1 — Supplementary figures. [file thnov11p7491s1.zip › Figure S3.pdf]

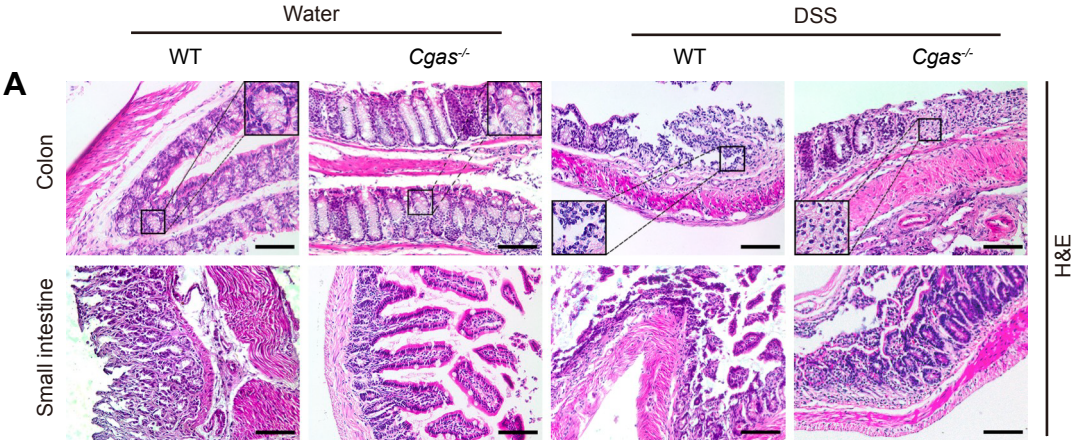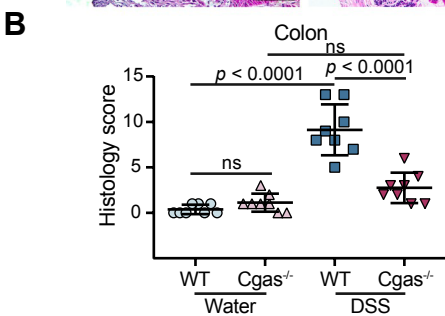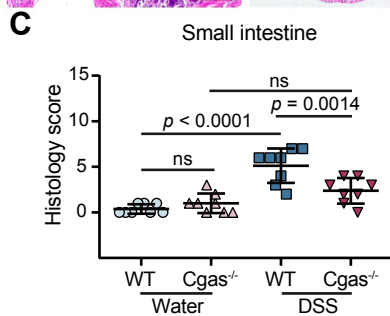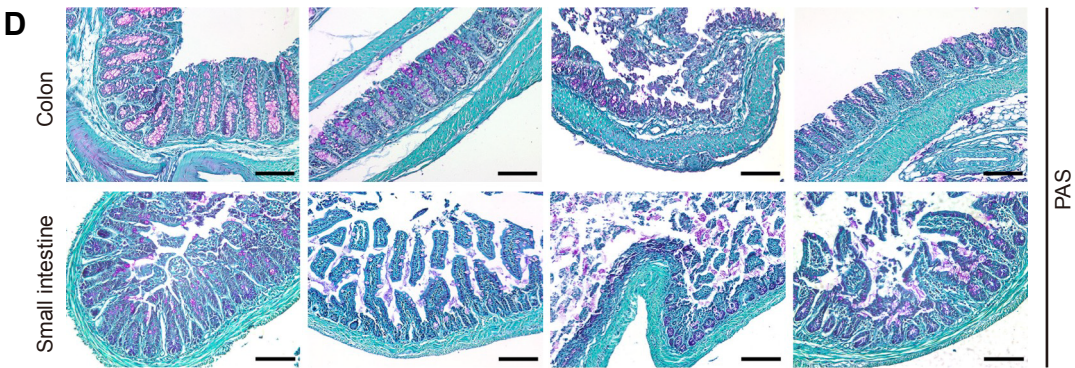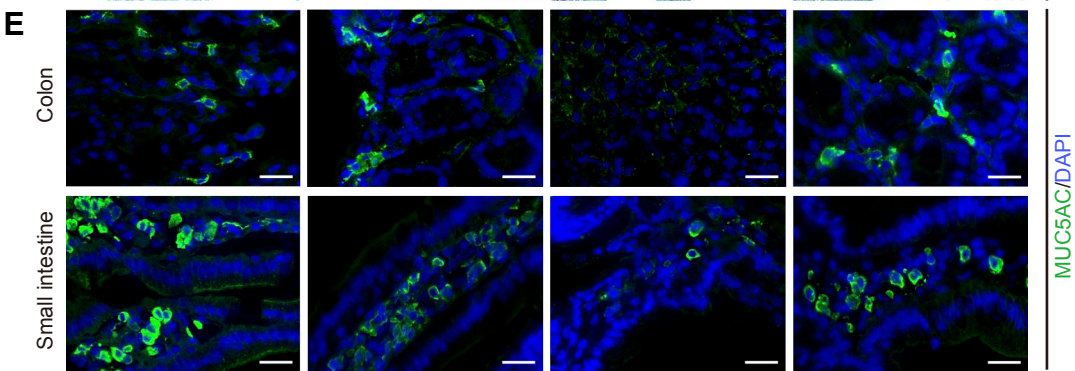

Supplement: Supplementary file 1 — Supplementary figures. [file thnov11p7491s1.zip › Figure S4.pdf]

**A**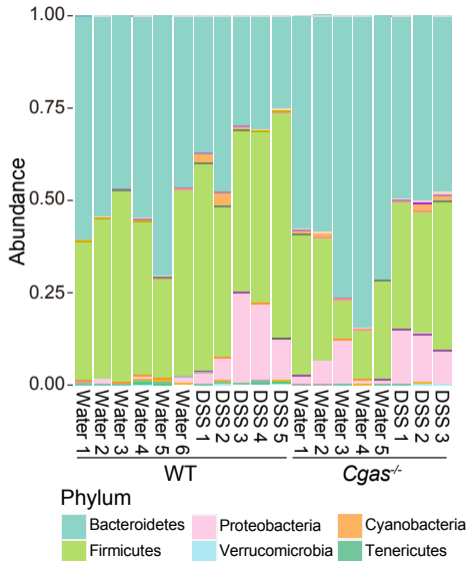**B**

WT+DSS vs. WT

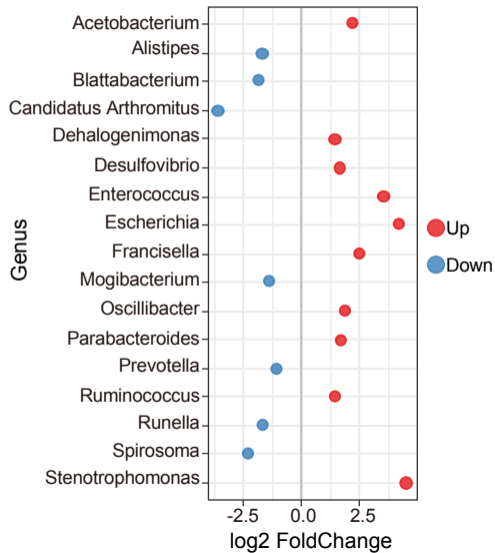**C***Cgas*<sup>-/-</sup>+DSS vs. *Cgas*<sup>-/-</sup>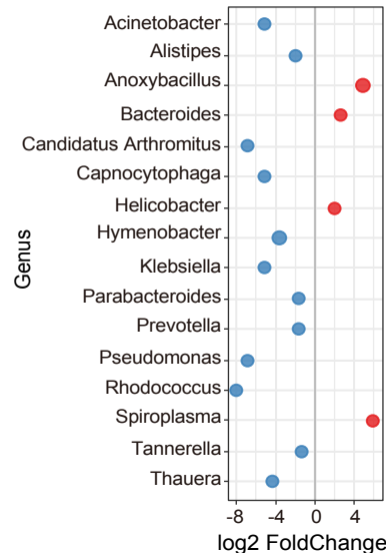

Supplement: Supplementary file 1 — Supplementary figures. [file thnov11p7491s1.zip › Figure S5.pdf]

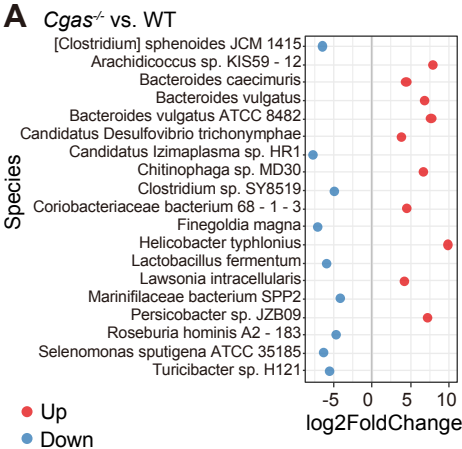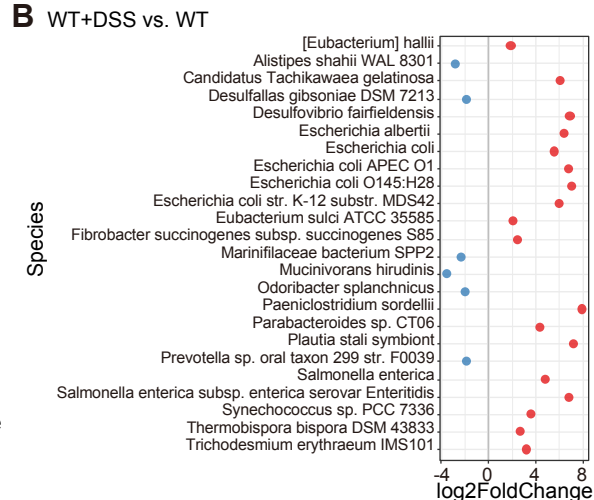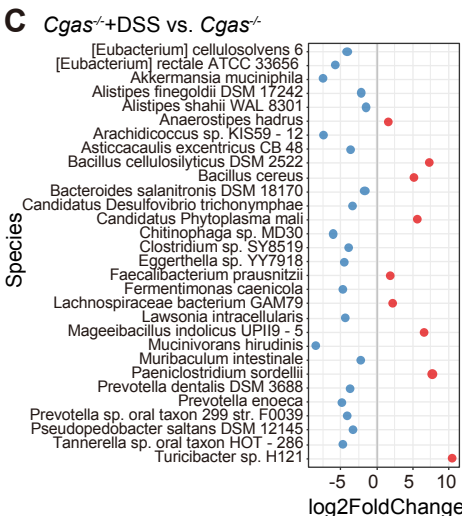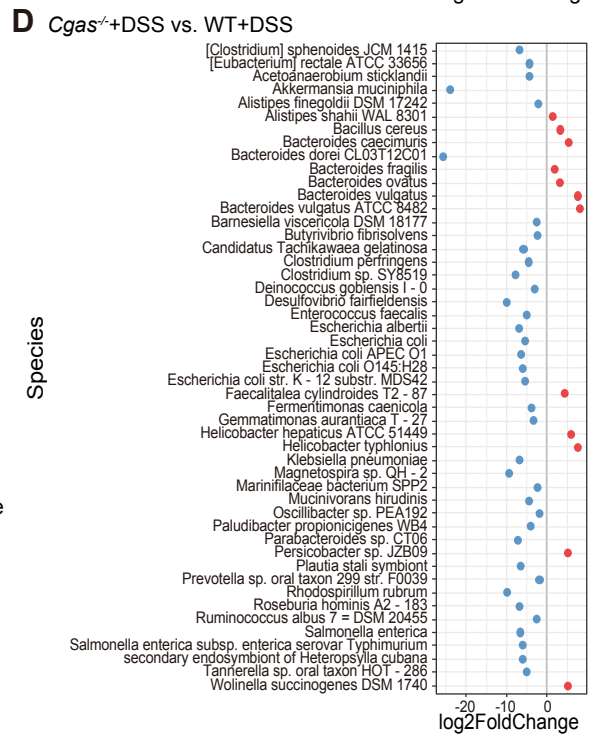

Supplement: Supplementary file 1 — Supplementary figures. [file thnov11p7491s1.zip › Figure S6.pdf]

**A**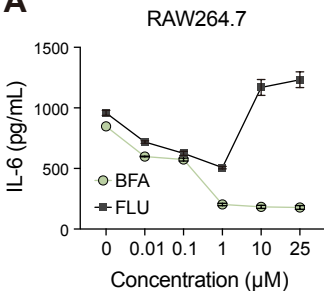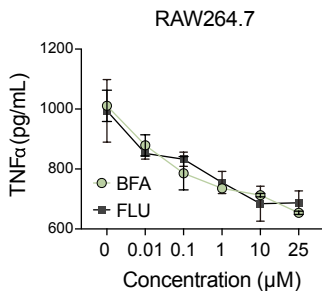**B**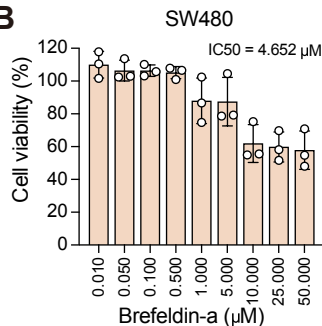**C**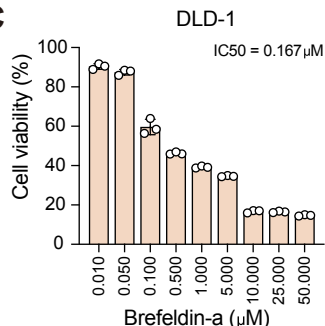**D**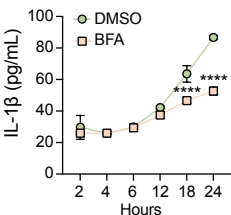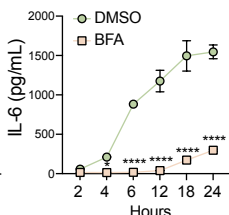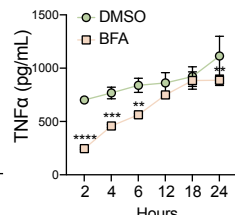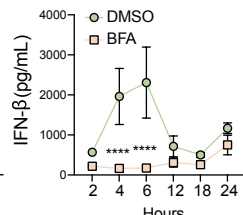**E**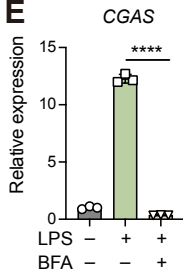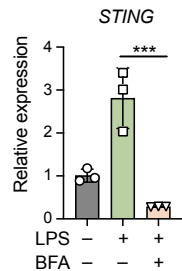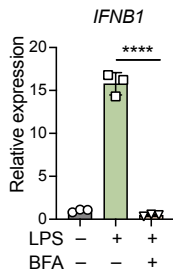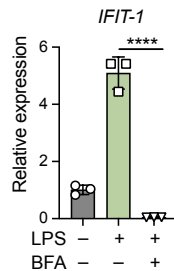**F**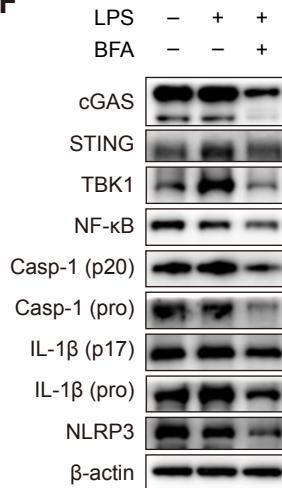

Supplement: Supplementary file 1 — Supplementary figures. [file thnov11p7491s1.zip › Figure S7.pdf]
